# Young Women's Stated Preferences for Biomedical HIV Prevention: Results of a Discrete Choice Experiment in Kenya and South Africa

**DOI:** 10.1097/QAI.0000000000001945

**Published:** 2019-01-09

**Authors:** Alexandra M. Minnis, Erica N. Browne, Marco Boeri, Kawango Agot, Ariane van der Straten, Khatija Ahmed, Rachel Weinrib, Carol Mansfield

**Affiliations:** a Women's Global Health Imperative, RTI International, San Francisco, CA;; b School of Public Health, University of California, Berkeley, CA;; c RTI Health Solutions, Research Triangle Park, NC;; d Impact Research and Development Organization, Kisumu, Kenya;; e Department of Medicine, Center for AIDS Prevention Studies, University of California, San Francisco, CA; and; f Setshaba Research Centre, Soshanguve, South Africa.

**Keywords:** discrete choice experiment, multipurpose prevention technology, HIV prevention, pregnancy prevention

## Abstract

**Background:**

Integrating end-user perspectives into the design of new biomedical HIV prevention products is recognized as vital to informing the product development pipeline.

**Setting:**

Kisumu, Kenya; and Soshanguve, South Africa.

**Methods:**

We conducted a discrete choice experiment survey with 536 women aged 18–30 years to assess preferences for hypothetical HIV prevention products characterized by the attributes of efficacy, pregnancy prevention, delivery form, dosing frequency, and side effects. Participants included product-experienced women from Tablet, Ring, Injection as Options TRIO, a cross-over clinical study evaluating 3 placebo delivery forms (oral tablets, vaginal rings, and injections), and a product-naive sample recruited from the same communities. Analyses used random parameters logit and latent class models.

**Results:**

HIV prevention efficacy was a strong determinant of stated choice overall; however, in South Africa, delivery form was just as important, with an injection every 2–3 months most preferred and a daily oral tablet least preferred. In Kenya, product-experienced women preferred monthly injections and least preferred a monthly ring. Respondents indicated a preference for multipurpose prevention technologies that combine HIV and pregnancy protection. Latent class analyses confirmed these findings and delineated heterogeneity in preferences across subgroups defined by age, past experience with the delivery forms, and education.

**Conclusions:**

Despite an overall preference for products with high efficacy, we identified attributes salient to future uptake and use of HIV prevention products. Preferences for injectable products underscored interest in this pre-exposure prophylaxis delivery form. Likewise, the multipurpose prevention technology feature was valued in both Kenya and South Africa and most influenced interest in vaginal rings.

## Abstract 2

Supplemental Digital Content is Available in the Text.

## INTRODUCTION

The HIV prevention field has increasingly recognized the importance of integrating end-user perspectives into the design of new biomedical HIV prevention products.^1,2^ Indeed, the ability to determine efficacy within large, randomized clinical trials and, ultimately, achieve successful uptake of effective products depends on end users' ability to initiate, persist with, and correctly use the product over time. With low adherence diluting the ability of HIV pre-exposure prophylaxis trials to determine product efficacy^3–6^ and diminishing the overall level of efficacy observed,^7^ there is an increased need to conduct rigorous end-user research earlier in product development to inform the product pipeline. Adolescent girls and young women in sub-Saharan Africa, a priority population at high risk of HIV, have been found to have lower adherence in studies of the vaginal ring and other delivery forms.^7–9^ Therefore, focusing end-user research on young women constitutes a priority to achieving and sustaining HIV prevention targets.^10^

Discrete choice experiments (DCEs), a behavioral economics methodology increasingly used to measure patient preferences for medical treatment features,^11,12^ engage respondents in considering a set of attributes that compose a potential product and then in making choices that indicate the attributes and tradeoffs most salient to future uptake and use. Increasingly, DCEs have been applied to inform HIV prevention, including preferences for HIV testing,^13,14^ pregnancy decision making in HIV-affected couples,^15^ and biomedical HIV prevention development.^16–19^ Studies of barriers to adherence in the context of trials of biomedical HIV prevention products highlight multiple attributes of products that shape users' experiences and willingness and ability to remain adherent over time,^8,20,21^ including factors such as delivery form, partner awareness of use, frequency of use, and side effects. Current open-label studies of oral pre-exposure prophylaxis inform understanding of key product features and contextual factors that may facilitate and diminish use of a known, active product.^22^

As part of a randomized, cross-over clinical study with 3 placebo products, the Tablet, Ring, Injection as Options (TRIO) Study,^23^ we conducted a DCE to examine attributes of a potential HIV prevention product influencing preferences among women at risk of HIV and unintended pregnancy in South Africa and Kenya. We assessed differences in preferences by geographic site and age. The design allowed us to examine preferences for HIV prevention products among women who had experience with the 3 placebo product delivery forms (vaginal ring, oral tablets, and injections), because they had participated in the TRIO clinical study, compared with those expressed by product-naive women recruited to complete only the DCE survey. Second, we explored the effect of delivery form on the probability that respondents would select one product profile over another when each form was assigned the most favorable attributes based on preferences estimated through the DCE. We examined how the probability of selecting a particular form shifted when a multipurpose prevention technology (MPT) feature for pregnancy prevention was added to the HIV prevention indication. Finally, using latent class (LC) analysis, we evaluated whether preferences varied between subgroups of women defined by sociodemographic and behavioral characteristics.

## METHODS

### Study Setting and Population

The TRIO DCE was conducted at 2 sites: Impact Research and Development Organization in Kisumu, Kenya, and Setshaba Research Centre in Soshanguve, South Africa. Details of the TRIO Study can be found elsewhere.^23–25^ In brief, TRIO consisted principally of a randomized, cross-over clinical study in which women aged 18–30 years tried 3 placebo products—vaginal rings, oral tablets, and injections for 1 month each and then selected one to use for the subsequent 2 months.^23^ The DCE component included 2 groups of participants: TRIO clinical study participants (“product-experienced”) and a newly recruited sample of women from the same communities who had not used the 3 delivery forms in the context of the TRIO Study (“product-naive”). Community-based, convenience sampling was used to recruit both participant groups. To enhance generalizability, the recruitment design aimed to draw a diverse sample of women residing in the communities in reasonable proximity to the research centers where the study was based. Community outreach teams convened informational community meetings in accessible locations during the week and on weekends to engage women in learning about the study as well as conducted door-to-door sensitization. These activities generated interest in the study and identification of potential participants who were then screened for eligibility. The TRIO clinical study participants completed the DCE survey at their final visit (5 months after enrollment). Based on the number of choice tasks, alternatives per task, and number of attribute levels, it was estimated that a sample size of at least 200 participants was needed per subgroup.^26^ Therefore, the target sample size for the DCE was 550 participants (250 in the product-experienced sample and 300 in the product-naive sample). Both sites received ethical and regulatory approvals before study initiation; all participants provided written informed consent.

### Development of the Discrete Choice Experiment

**FIGURE 1.**
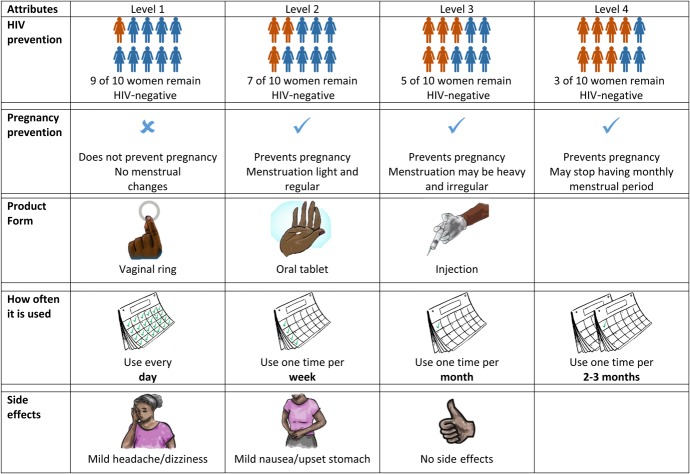
Discrete choice experiment design: Attributes and levels.

We conducted 30 in-depth interviews (15 per site) with women aged 18–30 years from the target population as formative research to inform selection of the attributes and their levels. Findings from each interview were summarized and synthesized for analysis. Interviews evaluated 14 candidate product attributes chosen for their potential influence on HIV prevention decisions based on our team's past research and review of the literature. Using a pile-sort approach adapted from participant attitudinal ranking,^27^ women rated the importance of each attribute to them in choosing a future HIV prevention product (as “very important,” “somewhat important,” or “not important”). The interviewer then probed using a semistructured interview guide to understand the rationale for how participants rated each attribute. In addition to synthesizing debriefing reports prepared by the interviewers that summarized these qualitative data, we calculated mean and median scores for each attribute, and ranked them by the number of participants who indicated the attribute was “very important,” to facilitate comparison. There was considerable alignment in the overall mean and median scores, and ranking, for each attribute at both sites. The attributes ranked as being most important included: HIV prevention efficacy; where to get the product; delivery form; frequency of use; and side effects. These rankings, alongside synthesis of the qualitative data and discussions regarding these attributes with the site teams and with providers in each community, shaped our final DCE design (Fig. 1). We also included an attribute for an MPT (with a range of menstrual side effects) that combined HIV and pregnancy prevention as it constituted a primary research objective of the TRIO Study.

Before finalizing the DCE design, we iteratively pretested the attribute descriptions and several choice questions derived from the selected attributes. During the pretests, we solicited input on the attribute descriptions, including their levels and the images developed to accompany the levels, to examine clarity of communication and cultural relevance. We included images for each attribute level to provide visual aids for lower literacy respondents. Second, we presented sample DCE choice questions to explore what influenced choices and decision making. Women involved in the formative research were excluded from participating in the DCE survey.

The experimental design for the DCE choice questions was created in NGENE 1.1 using a D-efficient algorithm to construct a fractional factorial experimental design.^28^ The design development followed good research practices.^29^ The full design included 48 choice questions that were divided into 6 blocks of 8 questions. Each respondent was randomly assigned to one block of 8 DCE questions.

### Study Design

Participants completed interviews on a tablet computer, with assistance from a research interviewer. The survey first introduced each attribute individually with both visual and narrative descriptions, and participants had to correctly answer a comprehension question regarding the graphics before continuing. Interviewers guided participants through each attribute description, pausing to ensure they understood each one before presenting the next one. Participants were then presented with 8 pairs of product profiles (choice sets) and asked to select their preferred product in each pair, making 8 unique choices (see Figure 1, Supplemental Digital Content, http://links.lww.com/QAI/B268). Following each choice, participants were asked whether they would prefer (1) the chosen product, (2) no protection, or (3) for their male partner to use condoms. This “opt-out” question was used to assess whether participants were “in the market” for an HIV prevention product and also whether the male condom was, in fact, preferred over the chosen product. After the choice sets, the survey also included direct-elicitation questions to assess the most important characteristics of an HIV prevention product. We measured participant sociodemographics, HIV risk, and sexual history.

### Analysis

Preference data are widely analyzed using random parameters logit (RPL) models,^30,31^ in which the sequence of the 8 choices among products is treated as the dependent variable and the attribute levels presented in the choice set are included as the independent variables. HIV prevention efficacy was modeled as linear as it met assumptions for linearity, whereas all other attributes included in the choice set were considered categorical and effect coded. With effect coding, zero indicates the mean effect across all attribute levels rather than the omitted level as in dummy coding.^32^ This procedure produces parameter estimates for all levels, where the parameter on the omitted level is the negative sum of the parameters on the included levels. Preferences for product form were expected to depend on the frequency of dosing (as some frequencies are relevant to only specific delivery forms—eg, daily dosing was presented for tablets only and not for rings and injections); therefore, models included an interaction term for frequency and delivery form. Because the sample reflected several distinct populations of participants (ie, 2 countries; product-experienced and product-naive samples within country), we tested for heterogeneity in preferences using the scale test procedure of Swait and Louviere.^33^ We estimated separate RPL models when differences were found.

We displayed RPL results graphically for ease of interpretation. The graphs present the mean preference-weight estimates for each attribute relative to the mean attribute effect, normalized around zero, with 95% confidence intervals. The weights indicate preference relative to other levels of the attribute, with larger positive numbers indicating greater preference and larger negative numbers indicating less preference. The preference weights for delivery form were combined with the results for frequency of use to present the interaction between frequency and delivery form. For each attribute, the difference between preference weights specifies the relative importance of moving from one level of the attribute to another. Although the magnitude of the weights can only be directly compared within each model, it is possible to compare, at least qualitatively, the relative importance of each attribute across models.

As a sensitivity analysis, we used LC modeling to explore heterogeneity in preferences. In contrast to RPL models, an LC model assumes preference heterogeneity after a discrete distribution, identifying for a specified number of underlying subgroups (classes) of participants with similar preferences. In addition to estimating preferences for different classes, the model provides the average membership probability for each class, where the probability of class membership is modeled as a function of respondents' characteristics. Separate LC models were estimated for Kenya and South Africa, with 2 classes comprising the optimal number in each, as determined using Bayesian Information Criteria. Class membership probability was estimated using the following characteristics hypothesized to influence preferences: age (18–24 vs. 25–30 years), education (attended university vs. not), sample (product-experienced vs. product-naive), own source of income, married or cohabiting, parity ≥1, multiple sexual partners in the past 30 days, important to use an HIV prevention product without partner's knowledge (vs. not), contraceptive method use (injectable, oral contraceptive pills, vaginally inserted products, and male condom), vaginal hygiene practices, and current use of daily medication. All models were estimated using NLOGIT software, version 5.0.

## RESULTS

The DCE survey was completed by 536 participants, 268 in Kenya and 268 in South Africa (56% product-naive at both sites); 96% of clinical study participants (all product-experienced) completed the DCE interview. Women's median age was 24 years, 94% had a primary partner, and 74% were parous (Table 1). Education level, marital or cohabitation status, and food insecurity varied by country.

**TABLE 1.**
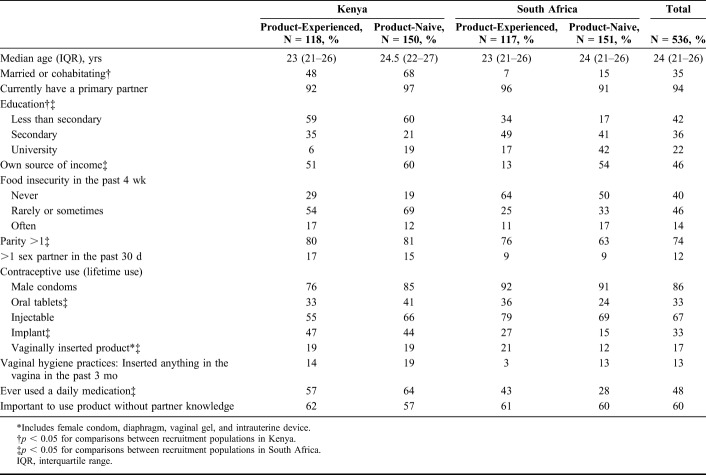
The TRIO Study Discrete Choice Experiment Participant Sociodemographic Characteristics by Country and Recruitment Population

### Preferences

We found significant differences in preferences between Kenya and South Africa (*P* < 0.001). In addition, in Kenya, we found a significant difference in preferences between the product-experienced and product-naive samples (*P* < 0.001). There were no significant differences by product experience in South Africa (*P* = 0.98). In addition, we found no differences in preferences between young women aged 18–24 years and those aged 25–30 years. Therefore, we conducted 3 separate analyses: 2 for Kenya (one each for the product-experienced and product-naive samples) and one for South Africa. Table 1, Supplemental Digital Content (http://links.lww.com/QAI/B268) contains the estimated preference weights from each model.

**FIGURE 2.**
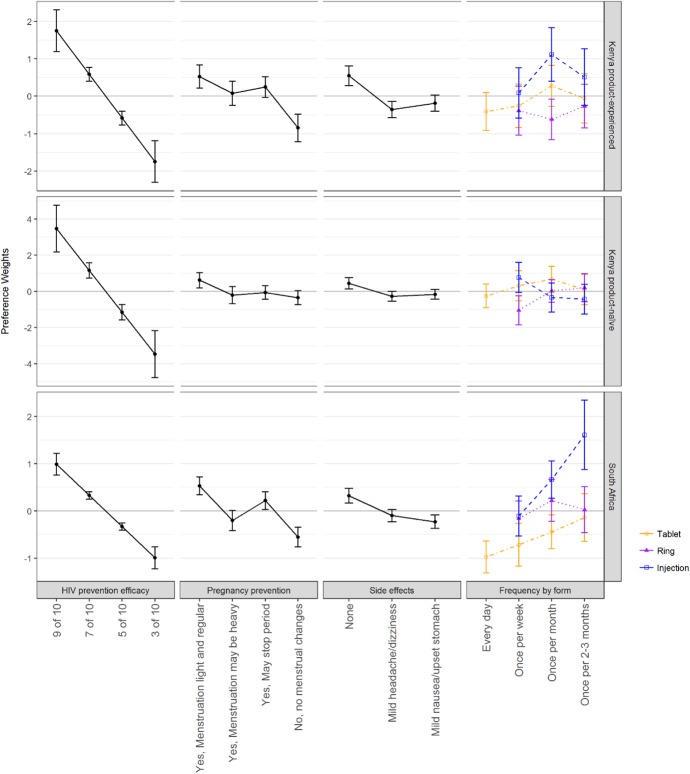
Normalized preference weights from random parameters logit models for Kenyan and South African women. Kenyan product-experienced and product-naive estimated separately due to preference heterogeneity.

Figure 2 displays the results from the 3 RPL models. Across all 3 populations, HIV prevention efficacy was a strong determinant of choice, as indicated by the vertical distance between the highest and lowest efficacy levels. Overall, for 20% of participants (n = 106), product choice was dominated by preference for HIV prevention efficacy, meaning for all 8 choice sets, these participants chose the product with the highest level of HIV protection. This attribute was particularly influential for the Kenyan product-naive sample, with 36% of participants (n = 55/150) for whom HIV prevention efficacy dominated all choices. In sensitivity analyses, no significant differences in preferences were found when removing these participants from the models.

In Kenya, both samples placed the most importance on HIV prevention efficacy, followed by pregnancy prevention (with lighter or regular menstruation) and no side effects. The Kenyan product-experienced sample also strongly preferred a monthly injection over a monthly vaginal ring (*P* = 0.002) and, across all dosing frequencies, tablets as a delivery form did not influence choice (*P* = 0.88). There was no statistically significant difference between the preference weights for product form in the Kenyan product-naive sample.

In South Africa, product form was as important as HIV prevention efficacy, with an injection every 2–3 months being the most preferred (*P* < 0.001). Women also preferred a product with pregnancy prevention (with lighter or regular menstruation), and no side effects. A daily oral tablet was the least preferred product form and frequency combination evaluated (*P* < 0.001).

### Preference Shares

**FIGURE 3.**
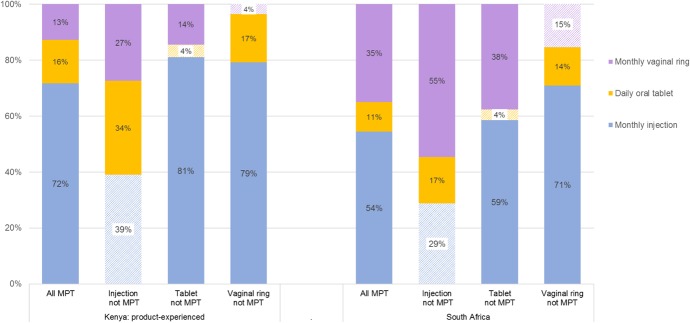
Preference shares for TRIO products among product-experienced Kenyan women and South African women. When all product delivery forms offer both HIV and pregnancy protection (are MPTs), a monthly injection received the highest share. However, when the monthly injection only prevents HIV but the vaginal ring and tablet remain MPTs, the preference shifts away from injection. All products had no side effects and provided 90% HIV protection. Hashed fill indicates product is not an MPT.

We used preference weights from the models to estimate the probability that the average respondent in each group would choose each of the 3 products used in the TRIO study if they were available: monthly vaginal ring, daily oral tablet, and monthly injection. As depicted in Figure 3, holding other attributes at their most desired level (no side effects, prevents pregnancy with light or regular menstruation, and 90% protection for HIV), monthly injection had the highest preference share in the Kenyan product-experienced sample (72%) and in the South African sample overall (55%). Because the Kenyan product-naive sample did not have a strong difference in preference based on product form, the probabilities of choice are relatively even across the 3 products (not shown). When the monthly injection is no longer an MPT, its preference share decreased to 29% in South Africa and to 39% in the Kenyan product-experienced sample. As depicted in the figure, this was accompanied by increases in the probability of choice for an MPT monthly vaginal ring (55% probability of choosing rings in South Africa and 27% in Kenya). Furthermore, although this resulted in only a modest increase in the probability of choosing tablets among the South African sample, the probability of tablet selection in the Kenyan sample increased from 16% to 34%.

### Use of Opt-Out After the Forced Choice Questions

The opt-out option (eg, prefer no new product or condoms) that followed the DCE choice question was selected in 15% of the DCE choice sets, highlighting that the choices of most respondents indicated an interest in a new type of HIV prevention product. For choices when the participant opted out, nearly all (97%) preferred that their partner use a condom over their chosen product, with the remaining 3% indicating they would use neither the product nor a condom. Seven percent (n = 35) of participants opted out of all 8 choice sets presented to them; the majority of those were from the Kenyan product-naive sample (n = 26).

### Latent Class Analysis

**FIGURE 4.**
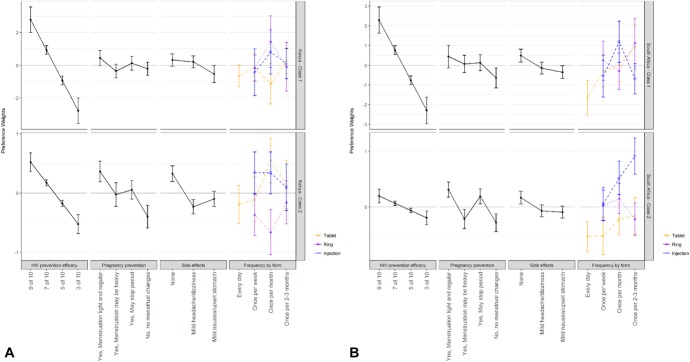
Normalized preference weights from latent class logit model of the South African sample (N = 268) and the Kenyan sample (N = 268). In Kenya (A, above), class membership was based on age and sample, with younger women (18–24 years) and those from the TRIO sample (product-experienced) more likely to be in class 2. In South Africa (B, above), class membership was differentiated based on education, with women who attended university more likely to be in class 1.

The LC modeling results are presented in Figure 4. In both countries, 2 subgroups of participants (or classes) were found to have distinguishing preferences. In Kenya, the 2 classes were characterized by age and study sample, with older women (aged 25–30 years) and those from the product-naive sample more likely to be in class 1, whereas younger women (aged 18–24 years) and those from the product-experienced sample more likely to be in class 2. Class 1 members (51% of the sample) had a very strong preference for HIV prevention efficacy, with no distinguishable preference across levels of other product attributes. Class 2, by contrast, was defined by a broader set of preferences, and preferred products with more HIV protection, that also prevented pregnancy, had no side effects, and were delivered as a monthly injection or monthly oral tablet vs. as a monthly vaginal ring. These results largely confirm those derived from the RPL analyses with heterogeneity in preferences between the product-naive and product-experienced samples.

In South Africa, classes were differentiated based on education, with women who attended university more likely to be in class 1 and those with less than a university education more likely to be in class 2. Class 1 members (34% of the sample) had a very strong preference for HIV prevention efficacy. However, they also valued the pregnancy prevention attribute and indicated a dislike for a daily oral tablet. Class 2 members also valued HIV protection, but this attribute was modestly influential to preference compared with class 1. They also preferred a product that prevents pregnancy (as long as it did not make menstruation heavier and irregular) and showed a strong interest in an injection every 2–3 months over a vaginal ring or oral tablets (at any dosing frequency). Both classes preferred a product with no side effects.

### Direct Assessment of Attribute Preference

Following the choice sets, participants indicated if the following characteristics are important when selecting an HIV prevention product: distribution location (60%), changes in menstrual cycle (50%), pregnancy prevention (80%), partner awareness (35%), weight gain (28%), and dosage frequency (57%). When asked which is the most important, 44% said pregnancy prevention, 22% chose distribution location, and 17% selected frequency of use. Participants in South Africa, compared with those in Kenya, were more likely to indicate that pregnancy prevention is the most important characteristic of product choice (59% vs. 30%, *P* < 0.001). Almost all participants (92%) preferred a “2-in-1” product that prevents both HIV and pregnancy compared with an HIV or prevention product only.

## DISCUSSION

The DCE findings highlight the overall importance of HIV prevention efficacy in influencing women's preferences for an HIV prevention product characterized by the attributes of efficacy, pregnancy prevention, delivery form, dosing frequency, and side effects. Yet, clear preferences for an HIV prevention product that also prevented pregnancy and that had no side effects were evident. Several attributes influential to preference varied between Kenya and South Africa and, within Kenya, between product-experienced and product-naive women. Product delivery form and frequency of use exerted a stronger role on preferences in South Africa than in Kenya, with South African women most preferring injections used every 2–3 months and expressing a dislike for tablets across all dosing frequencies. Product-experienced Kenyan women indicated preferences for a monthly injection and dislike for a monthly ring. Preferences of the product-naive sample in Kenya, however, were characterized, almost solely, by the level of HIV prevention efficacy. These findings align with those from previous choice experiments in South Africa that found high efficacy and integration of pregnancy prevention through an MPT to be important to demand for new HIV prevention products.^17–19^ This study extends previous work by comparing preferences in 2 distinct geographic sites, examining 3 product forms with distinct routes of delivery, focusing on younger women, and comparing DCE-elicited preferences in both product-experienced and product-naive women.

Differences in the product attributes that influenced preferences between the Kenyan and South African sites, and in the relative importance of specific attributes, highlight the variations in preferences regarding features of an HIV prevention product. The fact that HIV prevention efficacy was relatively more important in Kenya than in South Africa, and of paramount importance to preference for the product-naive sample of women in Kenya, may suggest a heightened perception of HIV risk in this group of women. The product-naive sample in Kenya may have prioritized HIV prevention efficacy above other attributes owing to a high desire to ensure that a chosen product “worked” to prevent HIV. Alternatively, it may be that current choices in other prevention areas (eg, contraceptives) are more constrained due to limited access and, therefore, the other attributes offered gained less traction as being pertinent to preferences. There may be underlying differences between the Kenyan and South African samples in opportunity to make choices among new health products and technologies. The premise, therefore, of making tradeoffs among attributes might have been weighed as less valuable by the product-naive Kenyan sample than selecting a product with high HIV prevention efficacy. Within Kenya, differences between the product-naive and product-experienced samples suggest that the experience of having used the products generated increased differentiation in preferences. The fact that this occurred with the Kenyan sample and not the South African sample may reflect the differences in educational levels at the 2 sites and the value, for the Kenyan women, of the direct opportunity to try the placebo products, better allowing them to form opinions about a broader range of attributes. It is indeed likely that community-level experience with new technologies, accompanied by tailored efforts to support users, will be needed to shape demand and identify user groups likely to adopt new products.

For women in South Africa and product-experienced women in Kenya, the preference share assessment that evaluated how the probability of product choice shifted when the MPT feature was (or was not) available in each form highlighted interest in an MPT product. Among the 3 product forms considered, an MPT vaginal ring is furthest along in development, with a phase 1 trial of a 3-month MPT ring completed (MTN-030/IPM 041). Thus, the finding that in South Africa, the estimated preference share for vaginal rings increased substantially when comparing it against an HIV-prevention only injection points to potential for an increased interest in and adoption of this less familiar delivery form when it offers dual protection. Likewise, the increase in the probability of choice of tablets among the Kenyan sample in scenarios when other products are not MPTs underscores the potential interest in MPT tablets.

### Limitations

Several limitations should be considered when interpreting results. Assessing preferences among a diverse set of products with varied delivery forms can be limited by preferences derived from hypothetical choice scenarios. What women expressed as their preferences may not ultimately align with actual adoption of HIV prevention due to multiple factors including relationship dynamics, access, and perceived risk. Although we adopted several design strategies to make the choices less hypothetical by anchoring them in previous experience with each attribute, we recognize that other contextual factors will ultimately contribute to women's choices. In addition, the preferences estimated through DCE are shaped by the attributes chosen, which are limited by what the methodology can accommodate to ensure the choices are not overly complex. Although the lack of a population-based sample does present some threats to generalizability, the community-based sensitization and outreach that accompanied the recruitment efforts, led by a well-established community outreach team at each of the local research organizations, was intended to achieve results that are robust and relevant to the communities from which participants were drawn. Although the period of adolescence and young adulthood is defined as extending to those aged 24 years, we elected to define our study population to include women aged 18–30 years to permit comparison between adolescent and young women with those close in age (25–30 years) who are also young and likely to be making decisions regarding both family planning and HIV prevention. Although we had envisioned, initially, presenting age-stratified analyses, we found no differences in preferences between these age groups. Furthermore, more in-depth analysis by age was not possible owing to small sample sizes, given the differences we did find by country and product experience subsamples (in Kenya). This finding of no age differences in preferences aligns with findings from the placebo clinical study conducted as part of our TRIO research activities.^23^ Given the importance of developing HIV prevention products that young people will adopt and use, and of understanding what aspects of a product will influence choice for various subgroups, future studies that focus on youth specifically and that are powered to explore differences within this age group could deepen understanding. Finally, efficacy proved to be such an important attribute that it may have diminished our ability to estimate preferences within levels of other attributes. In future applications of DCE methodology to HIV prevention research, it may be important to consider excluding efficacy to permit focus on other key attributes that will ultimately yield products favorable to end users.

## CONCLUSIONS

This DCE study, conducted in Kenya and South Africa, evaluated the attributes influential to young women's preferences for an HIV prevention product, informed by 3 delivery forms approved or in clinical studies: oral tablets, vaginal rings, and injections. Although women placed great value on a product with high efficacy, they also expressed preference for an MPT product that prevented both HIV and pregnancy. Indeed, although injections were estimated to have the highest preference share among the 3 delivery forms, and delivery form was the most important attribute for South African women, integrating pregnancy and HIV prevention in the ring increased its estimated share considerably. This was not the case for tablets. Thus, our findings underscore the benefit perceived by some women of an integrated MPT product and provide additional evidence of the potential for increased choice to achieve expanded adoption of biomedical HIV prevention by young women. Indeed, expanding options to achieve choice in HIV prevention tools is essential to reaching prevention goals. Preferences will vary between population subgroups and, likely, within individuals over time as sexual and reproductive health needs change.
